# Phenotypic Characterization of Subtype A and Recombinant AC Transmitted/Founder Viruses from a Rwandan HIV-1 Heterosexual Transmission Cohort

**DOI:** 10.3390/v16111706

**Published:** 2024-10-30

**Authors:** Ling Yue, Rui Xu, Samantha Mclnally, Qianhong Qin, Jake W. Rhodes, Erick Muok, Gisele Umviligihozo, Kelsie Brooks, Jiayi Zhang, Zhaohui Qin, Jean Bizimana, Jonathan Hare, Matthew A. Price, Susan A. Allen, Etienne Karita, Eric Hunter

**Affiliations:** 1 Emory Vaccine Center, Emory National Primate Research Center, Atlanta, GA 30329, USA; lyue2@emory.edu (L.Y.); xu-rui-rui@hotmail.com (R.X.); qianhong.qin@emory.edu (Q.Q.); jake.william.rhodes@emory.edu (J.W.R.); kelsie.brooks@nih.gov (K.B.); 2 Center for Family Health Research (Formally Project San Francisco), Kigali P.O. Box 780, Rwandagumviligihozo@rzhrg-mail.org (G.U.); jbizimana@rzhrg-mail.org (J.B.); ekarita@rzhrg-mail.org (E.K.); 3 Department of Biostatistics, Emory University, Atlanta, GA 30322, USA; jiayi.zhang2@emory.edu (J.Z.); zhaohui.qin@emory.edu (Z.Q.); 4 International AIDS Vaccine Initiative, New York, NY 10004, USAmprice@iavi.org (M.A.P.); 5 UCSF Department of Epidemiology and Biostatistics, San Francisco, CA 94158, USA; 6 Department of Pathology and Laboratory Medicine, Emory University, Atlanta, GA 30322, USA; sallen5@emory.edu

**Keywords:** infectious molecular clone, IMC, virus replicative capacity, co-receptor usage, bNAb potency and breadth

## Abstract

HIV-1 subtypes have distinct geographical distributions, with subtypes A, C, and D and inter-subtype recombinants circulating in sub-Saharan Africa. Historically, individuals living with subtype A viruses exhibit slower CD4 decline and progression to AIDS diagnosis. Despite this, there are few authentic infectious molecular clones (IMCs) of subtype A or AC recombinant transmitted founder (TF) viruses with which to investigate viral impacts on pathogenesis. In this study, we constructed 16 authentic subtype A1 and 4 A1C recombinant IMCs from the IAVI Rwandan Protocol C acute infection cohort and characterized these viruses phenotypically. The virus replicative capacity (RC) scores varied over 50-fold, but the natural substitution of non-consensus amino acids in the p17(MA) domain of Gag was generally linked to higher RC levels. Sensitivity to a panel of broadly neutralizing antibodies (bNAbs) showed that all but one TF was sensitive to N6, which targets the CD4 binding site, while bNAbs PG16 and PGT 128 had a similar level of potency but reduced breadth against our panel of viruses. In contrast, bNAb 10E8V4 revealed high breadth but much lower potency. This panel of well-characterized, authentic subtype A and AC recombinant IMCs provides a resource for studies on the role of the virus subtype in HIV-1 transmission, pathogenesis, and vaccine design.

## 1. Introduction

An estimated 38 million people worldwide are living with HIV, and two-thirds of these infected individuals live in Sub-Saharan Africa [1]. Even though more than half are receiving antiretroviral treatment, a significant fraction of treated patients is not virally suppressed [2,3], and HIV prevention remains a major problem in the fight against HIV. A global effort to design and develop an effective HIV-1 vaccine has been carried out over the last 30 years, but one of its major challenges is the enormous diversity of the virus.

HIV-1 has been classified into four phylogenetic groups—M, O, N, and P—based on nucleic acid sequencing of the viral genomic RNA, with group M being by far the most widespread [4,5]. Group M is subdivided into 10 different subtypes (A–D, F–H, J, K, and the newly identified L), with genetic variation between subtypes ranging from 20 to 35% depending on the genomic regions and the subtypes being compared [4,6,7]. Recombination between viruses of different subtypes is continually adding further diversity to the circulating strains [8,9,10,11,12]. To date, over 150 inter-subtype circulating recombinant forms have been described [13,14]. Therefore, to develop a broadly effective prophylactic vaccine, there is a clear need to gain insight into the genotypic and phenotypic features of the viruses from various geographic locations against which a potential vaccine must act.

We recently reported on the amplification and sequencing of near full-length single genomes (NFLGs) of viruses from the plasma of a total of 26 individuals with acute HIV infection from the Rwandan Protocol C heterosexual acute infection cohort and 21 individuals with recent infection from high-risk cohorts followed in government clinics in Kigali, Rwanda [15]. These genotypic data showed that while subtype A remains the dominant subtype, a significant fraction of infections were initiated by viruses that were recombinants of subtypes A and C, and that this fraction is continuing to increase over time [15].

Transmission of HIV from chronically infected individuals to their partners is quite inefficient and, in most cases, systemic infection is initiated by a single genetic variant, the transmitted founder (TF) virus, from the circulating quasispecies [16,17,18]. Indeed, very early in infection, during Fiebig stages I and II, the circulating virus is essentially clonal [17,19]. The application of near-full-length single-genome amplification and cloning has allowed the construction of authentic infectious molecular clones (IMCs) of the TF virus [20,21,22,23]. A majority of such clones represent subtypes B and C, with a much more limited number of subtypes A and D or AD and AC recombinants.

We report here on a panel of 20 Rwandan subtype A and AC recombinant IMCs most representing authentic TF viruses that have been phenotypically characterized in detail. We show that as with subtype C IMCs, the replicative capacity of viruses derived from the subtype A and AC recombinant clones varies by 50-fold, consistent with infectivity on TZM-bl cells. While all of the viruses were CCR5 tropic, they differed significantly in their sensitivity to a panel of broadly neutralizing antibodies. However, all but one was neutralized by the potent N6 antibody that targets the CD4 binding site. The availability of this panel of well-characterized subtype A and AC recombinant viruses will facilitate studies on the development of a potential HIV-1 vaccine.

## 2. Materials and Methods

### 2.1. Infectious Molecular Clone (IMC) Construction

As per our previous protocols [15,19], ~9 kb near-full-length single genomes were amplified from the plasma of 20 participants with acute infection and sequenced using PacBio DNA sequencing technology. Transmitted founder (TF) genomes were then identified or generated (Table 1).

Briefly, the entire authentic LTR (~650 bp) was amplified from each patient’s genomic DNA extracted from white cell pellets at the acute infection time point using patient-specific primers. IMCs were constructed by using TF amplicons [20] or synthesized sequence fragments of each virus [24] and each patient’s authentic LTR amplicons or sequences (vectors listed in Table 1).

### 2.2. Virus Stock Generation and Particle Infectivity

The 293 T cell line and Fugene-HD transfection reagent (Promega) were used to generate virus stocks [24,25]. Stocks were collected 48 h after IMC transfection. The titer of virus stocks was measured on the TZM-bl reporter cell line as described previously [24,26,27]. The virus stocks were also directly analyzed for reverse transcriptase (RT) activity using real-time PCR (described below). Particle infectivity of each virus stock was determined as the ratio of the virus titer (infectious units/μL) to RT activity (RT pg/μL) for 3 independent experiments [20,25].

### 2.3. Viral Replication Capacity Determination

Cryopreserved peripheral blood mononuclear cells (PBMCs) from a single healthy blood donor were used for viral replication assays. Infections were carried out using either whole PBMCs or CD4 T cells only. For infection with PBMCs, the cells were stimulated prior to infection with 20 U/mL of interleukin-2 (IL-2) and 3μg/mL of phytohemagglutinin (PHA) in 10% FBS RPMI (R10) containing 1 U/mL penicillin, 1 μg/mL streptomycin, and 300 μg/mL L-glutamine for 72 h at 37 °C and 5% CO_2_. Cells were infected at an MOI of 0.05, and supernatants were taken on days 1, 3, 5, 7, and 9 post-infection. Virus production at each time point was quantified using a P33-labeled reverse transcriptase assay as previously described [24,25,26]. Alternatively, for CD4 T-cell infections, the CD4 T-cell population was polyclonally expanded by incubating PBMCs in R10 with 50 units/mL IL-2 and 0.5 μg/mL CD3/CD8 bispecific antibody for 7 days [28]. Infections were carried out for 9 days at an MOI of 0.05, and supernatants were collected on the days described above. Viral replication was measured by RT activity at each time point using a real-time PCR assay (described below). The replication capacity (RC score) was determined by using the area under the curve calculated between day 3 and day 7 time points of the viral replication and then normalized against the area under the curve of wild-type MJ4 [26].

### 2.4. Reverse Transcriptase (RT) Activity Quantitation by Real-Time PCR

Accurate quantification of RT activity in the cell culture supernatant was used to measure virion production from infected CD4 T cells at each time point [29]. This method was an adapted version of the SG-PERT assay [30]. Briefly, standards were made using 10-fold serial dilutions of recombinant HIV Reverse Transcriptase (Life Technologies, Catalog# AM2045) in DMEM supplemented with 10% FBS and 0.5% BSA. Then, 5 μL samples (undiluted) and standards were aliquoted into 96-well U-bottom plates and incubated at a 1:1 ratio with lysis solution containing 2 × Lysis buffer composed of 0.25% Triton X-100, 50 mM KCL, 100 mM Tris HCL pH 7.4, 40% glycerol with 20 U RNAse inhibitor (Applied Biosystems, Foster City, CA Cat. #N8080119), and 1 mM DTT (Sigma–Aldrich, Burlington, MA) at room temperature for 10 min. The samples were then diluted with 40 μL ddH_2_O. qPCRs were performed in 96-well PCR plates (MicroAmp Optical 96-well reaction plate, Applied Bisosystems), with 4.75 μL virus–lysis solution and 5.25 μL qPCR master mix containing 1× SYBR Green I Master mix (Roche Diagnostics, Vilvoorde, Belgium; Cat. #04707516001), 20 U RNAse inhibitor, 0.1 μL MS2 RNA (1 mg/mL; Roche Diagnostics, Vilvoorde, Belgium, Cat. #10165948001), and 500 nM of both the MS2 FWD [5′-TCCTGCTCAACTTCCTGTCGAG-3′] and REV primers [5′-CACAGGTCAAACCTCCTAGGAATG-3′] (Integrated DNA technologies, Redwood City, CA). qPCR was performed using QuantStudio 3 (Applied Biosystems) under the following reaction conditions: 20 min (min) at 42 °C for RT reaction, 2 min at 95 °C for activation of FastStart Taq DNA polymerase, and 40 cycles of amplification as follows: 5 s (sec) at 95 °C for denaturation, 30 s at 60 °C for annealing and acquisition, and 15 s at 72 °C for elongation. The amount of MS2 cDNA synthesized from the RNA template correlates directly to the level of RT activity in each viral supernatant and the serially diluted RT standards. Through reference to the standard curve, it is possible to calculate the amount of reverse transcriptase enzyme in each sample and thereby a measurement of the number of retroviral particles [30].

### 2.5. Statistical Analysis

We conducted a comprehensive statistical analysis to evaluate the impact of specific amino acids at designated positions in the Gag protein sequences on RC values. Each sequence was split into individual amino acids, padded to a uniform length, and combined with corresponding RC values. Using the Wilcoxon rank sum test, we compared RC values between sequences containing the specified amino acids and those that did not. *p*-values from these tests were computed and reported. All data analyses were performed using R Version 3.6.1.

### 2.6. Sensitivity to Broadly Neutralizing Antibodies (bNAbs)

The sensitivity of viruses against a set of bNAbs (N6, VRC01, PG16, PGT 128, and 10E8V4, obtained from the NIH AIDS Reagent Program) was tested. Ten-fold serial dilutions were performed on each bNAb, resulting in test concentrations of 10 μg/mL, 1 μg/mL, 0.1 μg/mL, 0.01 μg/mL, and 0.001 μg/mL. bNAb neutralization assays were carried out in the TZM-bl cell line; residual infectivity was quantitated by measuring luciferase activity as described previously [31].

### 2.7. Coreceptor Usage

Maraviroc (CCR5 entry inhibitor) and AMD-3100 (CXCR4 entry inhibitor) were used at 2 μM to determine which coreceptors were utilized by the viruses. NL4.3 (CXCR4 tropic) and MJ4 (CCR5 tropic) were used as standards for infecting TZM-bl cells with entry inhibitors.

### 2.8. GenBank Submission

The GenBank accession numbers of the 20 IMC full-length sequences are JX236678, JX236677, MT942708, MT942722, MT942731, MT942736, MT942748, MT942773, MT942787, MT942802, MT942819, MT942836, MT942857, MT942878, MT942914, MT942927, MT942928, MT942941, MT942955, and PQ246051.

## 3. Results

### 3.1. Infectious Molecular Clone Construction

We generated 20 infectious molecular clones (IMCs), as described in the Methods, from viruses in the plasma of individuals with acute infection in a Rwandan heterosexual transmission cohort (IAVI protocol C) [15]. Samples were collected between the years of 2006 and 2011. The majority (19/20) of the clones were based on transmitted founder (TF) sequences derived from near full-length single-genome amplification and sequencing [15]. A total of 4 of the 20 IMCs were defined as A/C recombinants, and the remainder were entirely subtype A, as summarized in Table 1.

### 3.2. Phenotypic Analysis of IMC-Derived Subtype A and AC Recombinant Viruses

IMC-derived viruses were generated by transfection of 293 T cells, and virus stocks were harvested 48 h later. The virus titer was defined by infection on TZM-bl cells, a dual-reporter cell line [24,25].

**Figure 1:**
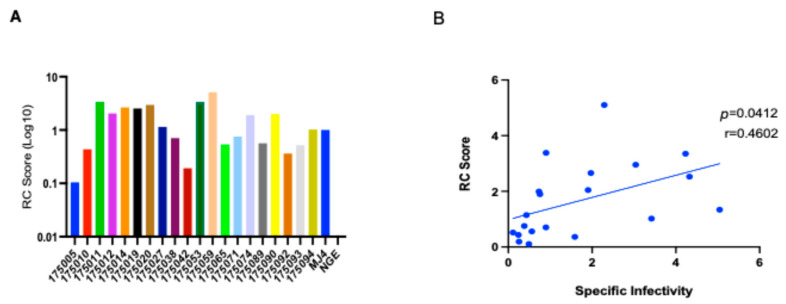
Replication and specific infectivity of IMC-derived viruses. (**A**) Replicative capacity (RC) score of IMC-derived viruses: defined by quantitating reverse transcriptase released into the culture medium and calculating the area under the curve from day 3 to day 7 post-infection, normalized by values for MJ4 (MJ4 = 1). (**B**) Correlation plot of specific infectivity and RC score (*p* = 0.041, r = 0.4602). Specific infectivity is the ratio of viral stock titer on TZM-bl cells to reverse transcriptase activity.

The replication capacity of these viruses was measured post-infection of PBMCs or CD4 cells, polyclonally expanded from PBMCs by bispecific anti-CD3/CD8 mAb [28], from a single healthy donor at a multiplicity of infection of 0.05. Post-infection supernatants were collected on days 1, 3, 5, 7, and 9, and virus growth was measured by quantitating RT activity at each time point by qPCR, as described in the Methods [29]. The virus replication capacity (RC) was calculated from the area under the curve of day 3 to day 7 post-infection RT values. To normalize across different experiments, an RC score was generated by normalization to the replication capacity of MJ4, as described previously [24,26]. The RC scores of these 16 subtype A and four unique AC recombinants in CD4+ T cells varied over 50-fold, with RC scores ranging from 0.1 to 5.1 (Figure 1A), as we have observed for subtype C TF viruses [25].

We determined the specific infectivity of each virus by dividing the virus titer on TZM-bl cells by the amount of reverse transcriptase (pg/mL) in the same sample. As with the RC scores, these specific infectivity values varied over 50-fold and were statistically correlated to their respective replicative capacities (Pearson correlation—*p* = 0.0412, r = 0.4602) (Figure 1B).

### 3.3. Non-Consensus Amino Acid Substitutions in Gag Increase Virus Replicative Capacity

Previous studies have demonstrated that TF viruses have sequences closer to subtype consensus or cohort consensus and that viruses with Gag proteins closer to cohort consensus have lower replicative capacity [25,26,32,33,34]. In an exploratory study, we compared each amino acid in the Gag of these 20 TF viruses to the Rwanda cohort consensus sequence to see how any of the natural changes from consensus affect replication capacity (Table 2). We found that amino acid changes at 13 positions in Gag significantly impacted the RC score (*p* < 0.05 Wilcoxon rank sum test). Most of the significant changes identified in this small number of viruses were in the p17(MA) protein.

In Table 2, we identified amino acids (column 2) at each of the 13 positions that were statistically associated with changes in replicative capacity. Most (positions 7, 9, 11, 20, 22, 47, 69, and 79) represented a single amino acid different from the consensus, but in the remaining five positions (30, 49, 62, 73, and 107), the consensus residue is highlighted since more than one amino acid substitution at each of these positions resulted in a significant change (increase) in replicative capacity. In position 49, for example, serine was substituted in six viruses by either glycine or aspartic acid, and these changes resulted in a 2.8-fold increase in RC relative to those viruses encoding the consensus residue. The majority (12/13) of these variants from the consensus were associated with higher RC scores; however, one at position 79, which involved the substitution of the highly hydrophobic phenylalanine for more polar tyrosine, was detrimental to replication (Table 2).

### 3.4. Broadly Neutralizing Antibody (bNAb) Potency and Breadth Against Subtype A and AC Recombinant Viruses

**Figure 2:**
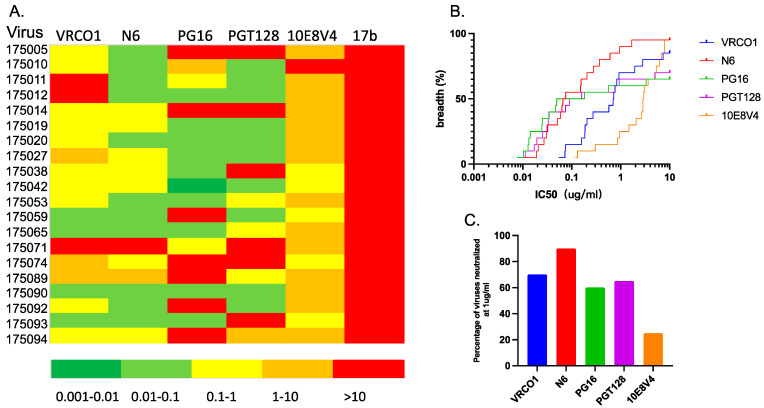
Sensitivity of IMC-derived viruses to a panel of broadly neutralizing antibodies (bNAbs). (**A**) The heatmap represents each IC50 value as the indicator of bNAb sensitivity. (**B**) Neutralization breath and potency curves of this panel of bNAb against the IMC-derived viruses. (**C**) Percentage of viruses neutralized by each bNAb at a concentration of 1 μg/mL.

Antibody-based vaccine development is exceptionally challenging because of the increasing genetic diversity of HIV-1 viruses [15,24,35]. This genetic diversity is also challenging for interventions aimed at using broadly neutralizing antibodies for HIV-1 prevention; measuring bNAb efficacy against different circulating viral subtypes and recombinants is crucial for this approach [36]. In order to characterize the phenotypes of the IMCs fully, we evaluated their sensitivity to bNAbs that target the major neutralization epitopes of the HIV envelope protein [V1V2-glycan apex (PG16), V3-glycan (PGT128), CD4 binding site (VRC01 and N6) and gp41 MPER (10E8V4)]. A non-neutralizing antibody, 17b, which binds a CD4-induced (CD4i) epitope on gp120 [37], was used as a non-specific antibody control. The 50% inhibitory concentrations (IC50s) used to measure bNAb sensitivities of all 20 IMC-derived viruses are shown in a heat map (Figure 2A). Previous studies reported that bNAbs targeting the CD4 binding site, especially antibody N6, achieved the most potency and highest breadth [38,39]. In this study, the CD4 binding site bNAb N6 also showed the most potency (median value = 0.07 μg/mL) and the greatest breadth against this panel of 20 IMC-derived viruses (95%) (Figure 2B,C).

**Figure 3:**
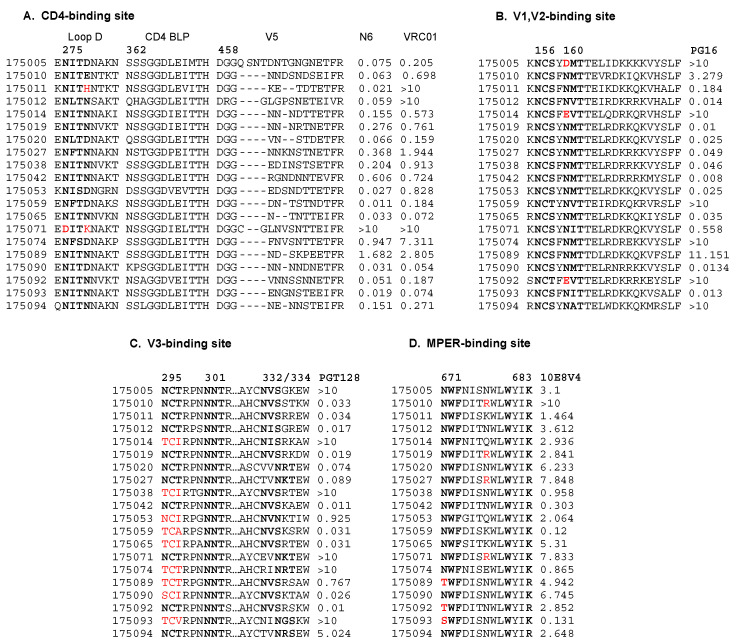
Major determinants of sensitivity to bNAb. (**A**) CD4 binding site. (**B**) V1,V2 binding site (**C**) V3 binding site. (**D**) MPER binding site. Amino acid sequences for each virus are shown for the sites on the left and IC50 on the right. Letters with red color indicate amino acid changes that are linked to significant bNAb resistance, and bolded letters signify key N-linked glycosylation sites.

Notably, N6 was both more potent and exhibited greater breadth than VRC01 (median potency = 0.71 μg/mL, coverage = 85%), which also targets the CD4 binding site epitope (Figure 2B,C). The determinants of resistance to CD4 binding site antibodies were previously defined as located in gp120 loop D, the CD4 binding loop, and the V5 region (Figure 3A) [39,40,41,42]. We also observed that in these subtype A or AC viruses, the N-linked glycosylation site at N276, previously shown to be involved in VRC01 binding to gp120 [39], was highly conserved in all but one virus. Similarly, at position 279 in loop D, where mutations have been identified as conferring resistance to N6, aspartic acid and asparagine predominate [43]. Of interest, virus 175071 has mutations at both positions (N276D and D/N279K) and is resistant to both N6 and VRC01 with an IC50 > 10 (Figure 3A).

This panel of subtype A and AC viruses is also quite sensitive to PG16 (median potency = 0.12 μg/mL) and PGT128 (median potency = 0.12 μg/mL) (Figure 2A). PG16 and PGT128 exhibit a breadth of 65% and 70%, respectively (Figure 2B), and the coverage of PG16 and PGT128 at 1 μg/mL is 60% and 65%, respectively (Figure 2C). The determinant region of PG16 resistance is centered on the N-glycan of gp120 N160, which is key for bNAb recognition [44,45]. In this panel of Rwandan IMC, viruses 175005, 175092, and 175014 all lack the N160-glycan and are highly resistant to the bNAb (IC50 > 10) (Figure 3B).

PGT128 targets an epitope in the V3 loop and four potential N-linked glycosylation sites, with N332/N334, N295, or N301 playing important roles in antibody susceptibility [46,47,48]. Previous studies summarized that there are two distinct routes for bNAb escape [49]. In this study, PGT128 was able to neutralize a majority of the viruses that encoded the glycan at N332 (78.6%) (Figure 3B). Viruses lacking N332 but encoding the adjacent N334 glycan require a glycan at N295 [49], and in this dataset, we observed that all but one of the N334/N295-encoding viruses are sensitive to PGT128; in contrast, viruses encoding N334 alone were highly resistant (175093, 175074; IC50 > 10) (Figure 3C).

Monoclonal antibody 10E8V4, targeting the gp41 MPER region [50], exhibits breadth but is only moderately potent. In this set of viruses, 10E8V4 showed equivalent breadth to N6, neutralizing all but one of the viruses (175010) but with lower potency (median IC50 = 2.89 μg/mL). At 1 μg/mL, only 20% of this panel of viruses were neutralized (Figure 2B,C). For 10E8V4, it was previously reported that the minimal epitope is within residues 671–683 (Figure 3D), where N671 and R/K 683 were critical for the binding; substitutions in 671–673, 680, and 683 also reduced sensitivity [50]. While these key amino acids were conserved in the majority of these subtype A or AC viruses (Figure 3D), we did not observe any consistent impact of changes at residue 671. Indeed, one of the most sensitive viruses (175093) had a serine in this position. At least for this set of viruses, residues outside the previously defined minimal epitope appear to modulate sensitivity to the antibody significantly.

### 3.5. Co-Receptor Usage Is Conserved in the Virus Panel

**Figure 4:**
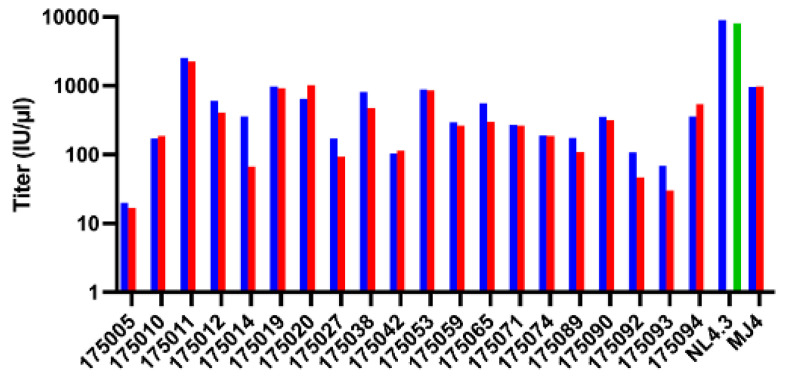
Coreceptor utilization of IMC-derived viruses. TZM-bl cells were infected in the absence or presence of 2 μM Maraviroc (CCR5 inhibitor) or AMD3100 (CXCR4 inhibitor). The bar graph shows infectivity with no inhibitor (blue bars), AMD3100 (red bars), and Maraviroc (green bars).

While most transmitted viruses utilize the CCR5 co-receptor [23,51,52,53], we utilized inhibitors of both CCR5 and CXCR4 to determine if this was also the case for the subtype A and AC recombinant viruses derived from the IMCs described here. All 20 of the viruses were inhibited completely in the presence of 2 μM maraviroc, while none were inhibited by 2 μM AMD-3100, consistent with CCR5 tropism (Figure 4).

## 4. Discussion

We previously amplified and sequenced the full-length viral genomes of 26 Rwandan subtype A and C transmitted founder and early viruses and defined the breakpoints for AC and CD recombinant viruses [15]. In this study, we generated infectious molecular clones (IMCs) from 20 of these sequences, the majority of which represented transmitted founder viruses, and performed a detailed examination of the phenotypic properties of IMC-derived viruses. Four IMCs (175027 TFp1,175071 TFp2, 175074 TFp1, and 175090 TFp1) were derived from instances where multiple viruses were transmitted, but a clear rake of highly conserved sequences could be identified (Table 1).

Quantitation of the replicative capacity in PBMCs or CD4+ T cells from a healthy donor showed that the virus panel has a range of replicative capacities similar to our studies of subtype C IMC-derived viruses [25,54]. In contrast to our previous study [55], we did not observe a significant difference between the RC of viruses derived from multiple founder virus infections and that of the panel as a whole, but this likely reflects the small number of viruses under study here.

Since we and others previously reported that RC was majorly influenced by Gag sequences [26,34,56,57,58,59], we performed an exploratory statistical analysis of the association between specific amino acids and RC. This confirmed our previous observation, even in this small dataset, that substitution of non-consensus amino acids in Gag was generally linked to higher RC levels. Interestingly, and consistent with our previous analysis of Gag-Pro-chimeric viruses [26], the majority of these mutations were localized to the p17(MA) domain (Table 2). As in a previous study, we also observed a correlation between the infectivity of viruses on TZM-bl cells and replicative capacity in PBMC/CD4, suggesting that viral entry is also a significant component of RC [25].

In this study, we found that entry of the subtype A and AC recombinant TF viruses was completely blocked by the anti-retroviral drug maraviroc, consistent with CCR5 utilization. In contrast, none of the viruses exhibited significant sensitivity to the CXCR4 inhibitor AMD-3100 (Figure 4). We also investigated the sensitivity of each of the viruses to a panel of broadly neutralizing monoclonal antibodies since such tools are currently being explored for prevention and cure approaches [60,61,62]. As was highlighted by the recent Antibody-Mediated Prevention studies, bNAb breadth and potency are key to the effectiveness of such an approach [60,63], and viral genetic diversity can dramatically impact this. Only a few subtype A [64] and no AC recombinant molecular clones have been created in the field since most previous studies used Env pseudotyped viruses to measure bNAb sensitivity. We measured all 20 IMC-derived viruses for their sensitivity to a panel of bNAbs that targeted the following major epitopes on the HIV-1 Env protein: the CD4 binding site (VRC01 and N6), the V1V2-glycan apex (PG16), the V3-glycan (PGT128), and the membrane–proximal external region of gp41 (MPER; 10E8V4). The majority of the viruses were sensitive to N6, with more than half exhibiting an IC50 of less than 100 ng/mL and only one exhibiting a resistance of >10 μg/mL. VRC01, which was used in the AMP study, on the other hand, exhibited similar breadth but had a much lower potency, requiring almost ten times the amount of antibody for neutralization compared with N6. PG16 and PGT128 showed similar potency to N6 but only for approximately 50% of the viruses in this panel. As has been reported previously on other virus panels, the 10E8V4 antibody showed significant breadth but only at much higher antibody concentrations [38].

Subtype A virus is widely spread in East African countries such as Rwanda, Kenya, and Uganda, while in Rwanda, AC recombinants are increasingly circulating in this geographic area as a new type of virus. Therefore, these well-characterized viruses will provide important tools for HIV vaccine development and studies on mucosal infection to eliminate transmission, as well as treatment.

## Figures and Tables

**Table 1:** IMC derivation, subtype, and cloning vectors.

PCID ^a^	EDI ^b^	VL ^c^	Sequence Type ^d^	Subtype	Cloning Strategy	IMC Vector
175005	14	2,090,040	TF	A1	TF amplicon	pBlue
175010	73	148,220	TF	A1	TF amplicon	pBlue
175011	50	184,270	TF	A1C	Synthesized Con	pUC57
175012	53	1,398,004	TF	A1C	TF amplicon	pBlue
175014	46	806,290	TF	A1	TF amplicon	pBlue
175019	10	3,000,000	TF	A1	TF amplicon	pBlue
175020	46	134,472	EV	A1	Synthesized	pUC57
175027	67	425,000	TF (P1)	A1	Synthesized TF	pUC57
175038	21	730,000	TF	A1	TF amplicon	pCR XL TOPO
175042	10	152,000,000	TF	A1	TF amplicon	pBlue
175053	15	1,876,000	TF	A1C	Synthesized TF	pUC57
175059	17	7,290,000	TF	A1	TF amplicon	pBlue
175065	14	31,700,000	TF	A1	TF amplicon	pBlue
175071	41	4,702,444	TF(P2)	A1	Synthesized TF	pUC57
175074	37	219,920	TF (P1)	A1	TF amplicon	pBlue
175089	13	223,440	TF	A1C	Synthesized TF	pUC57
175090	15	2,920,000	TF (P1)	A1	Synthesized TF	pUC57
175092	25	3,940,000	TF	A1	TF amplicon	pBlue
175093	15	3,760,000	TF	A1	TF amplicon	pBlue
175094	16	4,400,000	TF	A1	Synthesized TF	pUC57

^a^ PCID, protocol C identification number of the participant; ^b^ EDI, time in days since estimated date of infection; ^c^ VL, viral load in copies/mL at time of sample collection; ^d^ sequence type: TF—transmitted founder virus sequence amplicon identical to consensus; (P1) (P2): TF sequence identified in subpopulation 1 or 2 of multiple variants; EV: early virus isolate consensus; pBlue: pBluescript.

**Table 2:** Impact of non-consensus Gag amino acids on virus replicative capacity.

Codon	Amino Acid	Consensus Amino Acid	Sequences With/Without Residue	Sequences with Residue	Mean RC without Residue	Mean RC with Residue	*p*_Value
7	I	V	17	3	1.319853	2.987937	0.04035
9	R	S	18	2	1.370372	3.367312	0.04211
11	E	G	18	2	1.370372	3.367312	0.04211
20	K	R	18	2	1.370372	3.367312	0.04211
22	K	R	18	2	1.370372	3.367312	0.04211
30	R	R	4	16	2.827091	1.255809	0.02188
47	D	N	17	3	1.330663	2.926681	0.04035
49	S	S	6	14	2.823382	1.032931	0.01171
62	E	E	7	13	2.494512	1.072287	0.04556
69	Q	K	14	6	1.094515	2.679684	0.02002
73	E	E	2	18	3.367312	1.370372	0.04211
79	F	Y	11	9	2.185593	0.817755	0.00566
107	I	I	8	12	2.419958	1.003471	0.03871

## Data Availability

All data are available on request from the corresponding author.
